# Impact of Baseline ALBI Grade on the Outcomes of Hepatocellular Carcinoma Patients Treated with Lenvatinib: A Multicenter Study

**DOI:** 10.3390/cancers11070952

**Published:** 2019-07-07

**Authors:** Kazuomi Ueshima, Naoshi Nishida, Satoru Hagiwara, Tomoko Aoki, Tomohiro Minami, Hirokazu Chishina, Masahiro Takita, Yasunori Minami, Hiroshi Ida, Mamoru Takenaka, Toshiharu Sakurai, Tomohiro Watanabe, Masahiro Morita, Chikara Ogawa, Atsushi Hiraoka, Philip Johnson, Masatoshi Kudo

**Affiliations:** 1 Department of Gastroenterology and Hepatology, Kindai University Faculty of Medicine, 377-2 Ohno-Higashi, Osaka-Sayama 589-8511, Japan; 2 Takamatsu Red Cross Hospital, Takamatsu 760-0017, Japan; 3 Ehime Prefectural Central Hospital, Ehime 790-0024, Japan; 4 Department of Molecular and Clinical Cancer Medicine, University of Liverpool, Liverpool L69 3GE, UK

**Keywords:** hepatocellular carcinoma, ALBI grade, Child–Pugh score, lenvatinib

## Abstract

Background: This study investigated the impact of baseline liver function according to the Child–Pugh score and ALBI (albumin-bilirubin) grade on the outcomes of patients with unresectable hepatocellular carcinoma treated with lenvatinib. Methods: A total of 82 lenvatinib treated patients were included. The correlations of baseline liver function according to the Child–Pugh score and ALBI grade with treatment outcomes, including objective response rate per mRECIST (modified Response Evaluation Criteria in the Solid Tumor), time to treatment failure, treatment duration, and likelihood of treatment discontinuation due to adverse events, were assessed in patients with hepatocellular carcinoma treated with lenvatinib. Patients were divided into four groups: (1) Child–Pugh score 5 and ALBI grade 1 (group 1), (2) Child–Pugh score 5 and ALBI grade 2 (group 2), (3) Child–Pugh score 6 (group 3), and (4) Child–Pugh score ≥7 (group 4). Univariate and multivariate analyses were performed to identify the factors contributing to the objective response rate and likelihood of discontinuation due to adverse events. *Results:* Among the 82 patients analyzed, group 1 had the highest objective response rate (57.1%) and the lowest likelihood of treatment discontinuation because of adverse events (11.1%) among the four groups (*p* < 0.05 and *p* < 0.05). Multivariate analysis identified ALBI grade 1 and baseline AFP level <200 ng/mL as the significant predictors of a high objective response rate (*p* < 0.05 and *p* < 0.01), and confirmed that patients with ALBI grade 1 had the lowest probability of treatment discontinuation due to adverse events (*p* < 0.01). *Conclusions:* Patients with Child–Pugh score of 5 and ALBI grade 1 predicted a higher response rate and lower treatment discontinuation due to adverse events by lenvatinib treatment.

## 1. Introduction

Hepatocellular carcinoma (HCC) is one of the major causes of cancer-related death worldwide [1,2,3,4]. Currently, sorafenib and lenvatinib are the only targeted therapies approved for the first-line treatment of advanced unresectable HCC [5,6,7]. Sorafenib and lenvatinib is the standard of care for patients with advanced-stage (Barcelona Clinic Liver Cancer (BCLC) stage C) HCC with vascular invasion and/or extrahepatic spread, as well as for patients with intermediate-stage disease (BCLC stage B) HCC, who are refractory to transcatheter arterial chemoembolization (TACE) [8,9]. 

Lenvatinib is a multitargeted tyrosine kinase inhibitor of VEGF receptors 1–3, fibroblast growth factor receptors 1–4, PDGFRα, RET, and KIT [10,11,12,13]. A phase I study of lenvatinib in HCC showed that the maximum tolerable dose in patients with Child–Pugh class A and class B liver function was 12 mg and 8 mg QD, respectively [14]. In the phase III REFLECT study, lenvatinib met its primary endpoint by demonstrating a prolonging effect on overall survival (OS), as confirmed by statistical non-inferiority to sorafenib [7]. The median OS was 13.6 months in patients treated with lenvatinib and 12.3 months in those treated with sorafenib (hazard ratio (HR), 0.92; 95% confidence interval (CI), 0.79–1.06). Based on the results of the REFLECT study, lenvatinib was recently approved as a promising first-line agent for patients with unresectable HCC in Japan, the EU, the USA, and Asia, including China, Korea, and Taiwan.

Ascites and encephalopathy are slightly more subjective than the other three factors used to determine the Child–Pugh classification [15]. Also, albumin and ascites are confounding factors. The albumin-bilirubin (ALBI) score, which was recently developed, is calculated based on albumin and bilirubin concentrations, and is therefore simpler and more objective than the Child–Pugh classification system [16,17,18,19]. In addition, the ALBI score is based on continuous variables, allowing an accurate evaluation of liver function. Three ALBI grades, ALBI 1–3, are assigned according to the scores. The ALBI grade may be a more suitable measure of liver function for the treatment of HCC because liver function affects the maximum tolerable dose of systemic therapy agents in HCC. In the present study, we investigated the impact of ALBI grade and Child–Pugh score on the outcomes of patients with HCC treated with lenvatinib.

## 2. Methods

### 2.1. Patients

The present retrospective study included 82 consecutive patients who were treated with lenvatinib for unresectable HCC in the 2 institutions between July 2017 and December 2018. The inclusion criteria were as follows: Unresectable HCC confirmed by pathological or radiographic findings and followed up for more than 2 months after treatment initiation. The efficacy and safety of lenvatinib in patients with unresectable HCC were evaluated by stratifying patients according to ALBI grade and Child–Pugh score. Namely, patients were divided into four groups: (1) Child–Pugh score 5 and ALBI grade 1 (CP5A-G1) (n = 27), (2) Child–Pugh score 5 and ALBI grade 2 (CP5A–G2) (n = 19), (3) Child–Pugh score 6 (CP6A) (n = 30), and (4) Child–Pugh score ≥7 (CP-B) (n = 6). The study was approved by the ethics committee of each institute and complied with good clinical practice guidelines.

### 2.2. Treatment Protocol

Lenvatinib (Lenvima®; Eisai Co., Ltd., Tokyo, Japan) was administered orally to patients with unresectable HCC. The dose of lenvatinib was determined according to body weight and liver function: Patients weighing <60 kg with Child–Pugh A received 8 mg of lenvatinib once daily, whereas those weighing ≥60 kg received an initial dose of 12 mg of lenvatinib once daily, and those with Child–Pugh B received an initial dose of 8 or 4 mg of lenvatinib once daily. Starting with a reduced dose was permitted based on the patient’s condition. According to the guidelines for the administration of lenvatinib, the drug dose was reduced or the treatment was interrupted in patients developing ≥grade 3 severe adverse events (AEs) or any unacceptable grade 2 drug-related AEs. AEs were assessed using the National Cancer Institute Common Terminology Criteria for Adverse Events, version 4.0 [20]. This protocol was maintained until the symptoms resolved as indicated on the package insert.

### 2.3. Evaluation of Treatment Response

Treatment response was evaluated by dynamic CT in accordance with modified Response Evaluation Criteria in Solid Tumors (mRECIST) [21]. Tumor assessments were generally performed every 4 to 8 weeks. The objective response rate (ORR; complete response (CR) plus partial response (PR)), and disease control rate (DCR; CR, PR, plus stable disease (SD)) were assessed by investigators, according to mRECIST. 

### 2.4. Efficacy Outcome and ALBI Score

Dose reduction, interruption, and discontinuation due to AEs were assessed and compared according to liver function status. Time to treatment failure (TTF; time from the initial administration to treatment discontinuation for any reason, including disease progression, treatment toxicity, patient preference, or any cause of death) was estimated by the Kaplan–Meyer method. ALBI scores were calculated using serum albumin and bilirubin values as follows: (ALBI score = (log10 bilirubin [μmol/L] × 0.66) + (albumin [g/L] × −0.085)) [16,22]. 

OS (time from the initial treatment to any cause of death) and progression-free survival (PFS; time from the initial administration to progression or any cause of death) were also analyzed.

### 2.5. Statistical Analysis

Data are expressed as the mean and standard deviation or median and range. Statistical analyses were performed using Fisher’s exact test, the Kaplan–Meier method, and the log-rank test. Univariate and multivariate logistic regression analyses were performed to estimate the odds ratio for the correlation between baseline characteristics and ORR. A *p*-value of <0.05 was considered statistically significant. All analyses were performed using the SPSS Medical Pack for Windows, version 25 (SPSS, Inc., Chicago, IL, USA).

## 3. Results

### 3.1. Patient Characteristics

The characteristics of patients are summarized in Table 1. The median age was 71.5 years (21–92 years), and there were 59 men and 23 women. HCC was derived from HBV, HCV, and NBNC in 12, 37, and 34 cases, respectively. There were 34 patients with BCLC A or B and 48 patients with BCLC C disease.

There were 29 patients (35.4%) with ALBI grade 1 and 52 patients (63.4%) with ALBI grade 2. Most of the patients with ALBI grade 1 (27 of 29 patients: 93.1%) had a Child–Pugh score of 5. Patients with ALBI grade 2 included 19, 28, and 5 patients with Child–Pugh scores 5, 6, and ≥7, respectively. One patient in the current study was classified as ALBI grade 3. The median scores corresponding to ALBI grades 1 and 2 were −2.73 (range, −3.10 to −2.64) and –2.14 (range, −2.57 to −1.45), and the median albumin levels of patients with ALBI grades 1 and 2 were 4.0 (range, 3.7–4.4) and 3.4 (range, 2.7–4.2). The median number of previous TACE procedures was one in the CP5A-G1 group, two in the CP5A-G2 group, and four in the CP6A group.

At the data cutoff date (December 31, 2018), 45 patients (54.9%) remained on the treatment regimen. Treatment was terminated in 37 patients (45.1%); 10 patients (12.2%) discontinued treatment because of PD, whereas 24 patients (29.3%) discontinued because of AEs during the follow-up period (Table 2). The median follow-up period was 82.5 days (range, 2–361 days). 

### 3.2. ORR and DCR According to ALBI Grade and Child–Pugh Score

The median PFS was 7.6 months (95% CI, 4.3–not reached) in the entire cohort. The ORR was evaluable in 59 of 82 patients at the data cutoff. The ORR was 39.0% (23/59), and the DCR was 83.1% (49/59) (Table 2). The ORRs according to the ALBI grade and Child–Pugh score are summarized in Table 2. The ORR in Child–Pugh A patients was 41.8%, consistent with the results with the REFLECT trial, whereas 0% in Child–Pugh B liver function. The ORR (57.1%) was significantly higher in the CP5A-G1 group as compared with ORR in the CP5A-G2 group (38.5%), CP6A group (28.6%), and CP-B group (0%), respectively. (*p* < 0.05). There were 32 patients who experienced sorafenib and 12 patients who experienced regorafenib. The ORR to lenvatinib after sorafenib as a second line treatment was 31.2% and that after regorafenib as a third line treatment was 16.7%, probably because of impaired liver function.

### 3.3. Dose Reduction, Interruption, and Discontinuation due to AEs according to Liver Function Status

The CP5A-G1 group showed the lowest likelihood of dose reduction and interruption due to AEs (48.1%) and treatment discontinuation because of AEs were also lowest (11.1%) among that in the CP5A-G2 group (26.3%), CP6A group (33.3%), and CP-B group (100%), respectively (*p* < 0.05) (Table 3).

### 3.4. TTF and Duration of Treatment According to ALBI Grade and Child–Pugh Score

**Figure 1:**
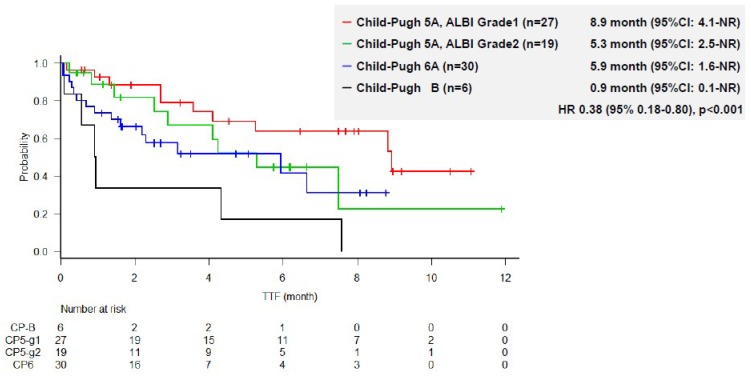
Time to treatment failure according to liver function.

The median TTF was 4.2 months (95% CI, 2.7–7.5). Kaplan–Meier estimates of TTF according to ALBI grade and Child–Pugh score are shown in Figure 1. The median TTF was 8.9 months (95% CI, 4.1-NE months) in patients with CP5A-G1 (n = 27), 5.3 months (95% CI, 2.5–NE months) in patients with CP5A-G2 (n = 19), 5.9 months (95% CI, 1.6-NR months) in those with Child–Pugh 6A, and 0.9 months (95% CI, 0.1-NR months) in those with CP-B (n = 6) (Figure 1). Liver function according to the ALBI grade and Child–Pugh score was significantly associated with TTF; patients with CP5A-G1 had the longest TTF, whereas those with Child–Pugh B had the shortest TTF (*p* < 0.01).

**Figure 2:**
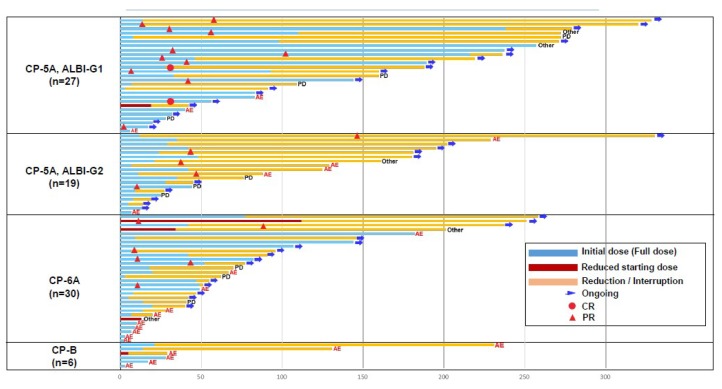
Duration of treatment according to liver function (n = 82).

The duration of treatment according to liver function is summarized in Figure 2. The median dose intensity and relative dose intensity were 8.4 mg/day, 84.4% and 7.5 mg/day, 97.5% for the group with body weight >60 kg and <60 kg, respectively. Also, 11.5 mg/day, 100% for body weight >60 kg and 7.7 mg/day, 96.8% for body weight <60 kg in patients with CP5A-G1. The responses were robust and durable in all groups, especially in the CP5A-G1 group. In 18 of 27 (66.7%) patients with CP5A-G1, lenvatinib treatment was still ongoing at the data cutoff. The responses were robust and durable especially in patients with CP5A-G1 liver function. Only 3 of 27 (11.1%) patients discontinued treatment because of AEs (Supplementary Table S1).

### 3.5. Univariate and Multivariate Regression Analysis

Univariate logistic regression analysis of baseline factors affecting ORR was performed for the following factors: Age, hepatitis B status, hepatitis C status, number of prior TACE procedures, ALBI grade, baseline α-fetoprotein (AFP) level, extrahepatic spread, and macrovascular invasion. ALBI grade 1, and baseline AFP level <200 ng/mL showed *p*-values ≤0.10 in the univariate analysis. Multivariate analysis identified ALBI grade 1 and baseline AFP level <200 ng/mL as independent predictors of ORR (*p* < 0.05 and *p* < 0.01 respectively) (Table 4).

Univariate logistic regression analysis of baseline factors affecting drug discontinuation because of AEs was performed for the following factors: Age, hepatitis B status, hepatitis C status, numbers of prior TACE procedures, ALBI grade, baseline AFP level, extrahepatic spread, and macrovascular invasion. ALBI grade showed *p*-values <0.01 in univariate analysis. Multivariate analysis confirmed that the rate of drug discontinuation because of AEs was significantly lower in patients with ALBI grade 1 than in those with ALBI grade ≧2 (odds ratio, 0.22 (95% CI, 0.06–0.69), *p* < 0.01) (Table 5). 

OS in patients with ALBI grade 1 was significantly better than that in those with ALBI grade 2 (HR = 0.12 (95%CI 0.02–0.97), *p* < 0.01) (Supplementary Figure S1).

## 4. Discussion

There is a wide variety of treatment outcomes (survival, ORR, treatment duration, or AEs, etc.) among patients receiving lenvatinib and at present, there is little evidence as to what factor accounts for these differences or how to predict them. In the present study, patients with CP5A-G1 had better outcomes than those in the other groups. Treatment duration and TTF were significantly longer in the CP5A-G1 group than in the other groups. ALBI grade 1 and low AFP level <200 ng/mL at the start of lenvatinib therapy was a useful predictor of a high ORR, and ALBI grade 1 at the start of lenvatinib therapy was the only factor predicting a low rate of treatment discontinuation because of AEs. 

The Child–Pugh classification is the most commonly used scale to evaluate liver function in clinical practice; however, it contains several less objective or measurable factors, such as ascites and hepatic encephalopathy, as compared with the other three factors. In addition, ascites and albumin are confounding factors [15]. By contrast, the ALBI score is based on only two factors, serum albumin and bilirubin levels, and is simpler and more objective than the Child–Pugh classification system. In addition, the ALBI grade varies among patients with the same Child–Pugh score. The Child–Pugh score can be further divided into ALBI grades 1 and 2. Among 22,098 patients classified as Child–Pugh score 5 according to the nationwide survey of the Liver Cancer Study Group of Japan, 14,418 (62.3%) had ALBI grade 1 and 7680 (33.2%) had ALBI grade 2 [23]. In addition, the ALBI grade is suggested to be a more sensitive measure to identify better liver function than the indocyanine green retention test at 15 m (ICG R15) or the Child–Pugh score [17,24,25]. In other words, a Child–Pugh score of 5 split into ALBI grade 1 and 2, and liver function is better in CP5A-G1 than that in CP5A-G2 [26]. 

In the present study, the CP5A-G1 group showed better tolerability and included a greater number of patients who maintained the initial dose than the other groups, resulting in the highest ORR. The rate of drug discontinuation because of AEs was low, and the management was relatively easy in this group of patients. However, most of the patients in the current study population were categorized as ALBI grade 2 or a Child–Pugh score of ≥6. In patients with CP5A-G2, AEs were relatively manageable and allowed continuation of lenvatinib treatment; however, maintaining the initial dose was difficult and dose reduction was frequently necessary. In patients with a Child–Pugh score of 6, earlier dose reduction and dose interruption were required to continue lenvatinib treatment. Generally, lenvatinib is indicated for patients with Child–Pugh class A liver function. However, in the present study, patient outcomes differed significantly according to Child–Pugh score and ALBI grade status. The clinical relevance of this finding is that, even among patients with Child–Pugh grade A, baseline liver function measured by the ALBI score plays an important role in the prediction of patient outcomes.

Another important finding in the current study is that the preservation of liver function was associated with a fewer number of previous TACE sessions. In the present study, the CP5A-G1 group, CP5A-G2 group, CP6A group, and CP-B group received prior TACE procedures in 1, 2, 4, and 7 times. Accordingly, the ORRs in these four groups were 57.1%, 38.5%, 28.6%, and 0.0%. Similarly, the dose reduction or interruption rate and discontinuation rate of lenvatinib tended to be lower in accordance with liver function status. Considering that the number of prior TACE procedures is closely related to liver function deterioration, it might be true that less prior TACE procedures correlates to a higher ORR and a lower discontinuation rate of lenvatinib. Similarly, a previous study reported that, at 3 months after each session of TACE procedures, the Child–Pugh score of patients originally classified as Child–Pugh A increased to 7 or more in more than 10% of patients in each session [26]. In addition, the ALBI grade became significantly worse in association with repeated TACE procedures [26]. Patients receiving repeated TACE showed a greater increase in Child–Pugh score than those who switched to sorafenib among patients with HCC who were deemed refectory to TACE [27,28]. Previous studies together with the present findings suggest that repeated TACE procedures appear to impair liver function. Thus, an early switch to systemic therapy, such as lenvatinib, from TACE may be important to maintain liver function and avoid dose reduction/interruption due to AEs, which should lead to improvements in the overall outcome, including ORR and OS. 

## 5. Conclusions

In conclusion, the present study demonstrated the effects of baseline liver function measured by the Child–Pugh score and ALBI grade on the efficacy and adverse event outcomes of patients with unresectable HCC treated with lenvatinib. This is the first study reporting that the objective response, frequency of adverse event, and treatment duration caused by lenvatinib treatment is closely related with liver function, especially with ALBI grade. The results indicated that a Child–Pugh score of 5 with ALBI grade 1 may predict a longer treatment duration and better outcomes of lenvatinib treatment. Thus, lenvatinib treatment should be started at an early phase, when liver function is preserved within ALBI grade 1, instead of repeating ineffective and/or liver function impairing procedures, like unselected TACE.

## Figures and Tables

**Table 1:** Patient characteristics.

Parameter	All Patients (n = 82)	
Median age, years (range)		71.5 (21–92)
Sex, n	Male/Female	59/23
Body weight, n	<60 kg/≥60 kg	36/46
Etiology, n	HBV/HCV/nonBnonC	12 */37 */34
Starting dose, n	12 mg/8 mg/4 mg	37/40/5
Median ALB (g/dL)		3.6 (2.5–4.4)
Median BIL (mg/dL)		0.7 (0.2–2.9)
Median PT (%)		88.0 (14.1–130.0)
Child–Pugh score	5/6/≥7	46/30/6
ALBI Grade	1/2/3	29/52/1
BCLC stage	A/B/C	7/27/48
Median AFP (ng/mL)		106.8 (1–4,503,000)
Median DCP (mAU/mL)		878 (8–538,430)
Median AFP-L3 (%)		20.9 (0–99.5)
Sorafenib	Naïve/Experience	50/32
Regorafenib	Naïve/Experience	70/12
Median number of previous TACE	Child–Pugh A/Child–Pugh B	2/7
Median number of previous TACE	Child–Pugh 5A, ALBI Grade1/Child–Pugh5A, ALBI Grade2/Child–Pugh 6A	1/2/4

* One patient is HBV positive + HCV positive etiology; ALB: albumin, BIL: bilirubin, PT: prothrombin time, AFP: alfa-fetoprotein, DCP: des-gamma-calboxy protein.

**Table 2:** Objective response rate (n = 59).

Liver Function	n	CR	PR	SD	PD	ORR	DCR
Child–Pugh A	55	2	21	24	8	41.8%	85.5%
5A, ALBI Grade1	21	2	10	8	1	57.1%	95.2%
5A, ALBI Grade2	13	0	5	5	3	38.5%	76.9%
6A	21	0	6	11	4	28.6%	81.0%
Child–Pugh B	4	0	0	2	2	0.0%	50.0%
Total	59	2	21	26	10	39.0%	83.1%

CR: complete response, PR: partial response, SD: stable disease, PD: progressive disease, ORR: objective response rate, DCR: disease control rate.

**Table 3:** Dose reduction, interruption, and discontinuation rate due to adverse event.

Liver Function	n	Reduction or Interruption Due to AE		Discontinuation Due to AE	
Child–Pugh A	76	47	61.8%	16	21.1%
5A, ALBI Grade1	27	13	48.1%	3	11.1%
5A, ALBI Grade2	19	15	78.9%	5	26.3%
6A	30	19	63.3%	10	33.3%
Child–Pugh B	6	2	33.3%	6	100.%
Total	82	49	59.8%	22	26.8%

**Table 4:** Univariate and multivariate logistic regression analysis for factors affecting ORR.

Parameters	Univariate Analysis		Multivariate Analysis	
	Odds Ratio (95% CI)	*p*	Odds Ratio (95% CI)	*p*
ALBI grade				
Grade 1	2.64 (0.81–8.97)	*p* < 0.1	3.32 (1.04–10.50)	*p* < 0.05
Grade ≥2	1		1	
Baseline AFP level				
AFP < 200 ng/mL	2.89 (0.90–9.90)	*p* < 0.1	3.58 (1.14–11.30)	*p* < 0.01
AFP ≥ 200 ng/mL	1		1	

**Table 5:** Univariate and multivariate logistic regression analysis for factors affecting the treatment discontinuation due to AE.

Parameters	Univariate Analysis		Multivariate Analysis	
	Odds Ratio (95% CI)	*p*	Odds Ratio (95% CI)	*p*
ALBI grade				
Grade 1	0.19 (0.03–0.76)	*p* < 0.01	0.22 (0.06–0.69)	*p* < 0.01
Grade ≥2	1		1	

## Supplementary Materials

The following are available online at https://www.mdpi.com/2072-6694/11/7/952/s1, Figure S1: Kaplan–Meier estimates of OS in patients treated with lenvatinib. According to the ALBI Grade, Table S1: Discontinuation rate of lenvatinib according to each group.
