# Supplementary material for: Impact of Baseline ALBI Grade on the Outcomes of Hepatocellular Carcinoma Patients Treated with Lenvatinib: A Multicenter Study
Source: Cancers (Basel). 2019 Jul 7;11(7):952. doi: 10.3390/cancers11070952 (PMC6678474; doi:10.3390/cancers11070952)
Supplement: Supplementary file 1 [file cancers-11-00952-s001.pdf]

Supplementary Material

Impact of Baseline ALBI Grade on the Outcomes of Hepatocellular Carcinoma Patients Treated with Lenvatinib: A Multicenter Study

Kazuomi Ueshima, Naoshi Nishida, Satoru Hagiwara, Tomoko Aoki, Tomohiro Minami, Hirokazu Chishina, Masahiro Takita, Yasunori Minami, Hiroshi Ida, Mamoru Takenaka, Toshiharu Sakurai, Tomohiro Watanabe, Masahiro Morita, Chikara Ogawa, Atsushi Hiraoka, Philip Johnson and Masatoshi Kudo

Table S1. Discontinuation rate of lenvatinib according to each group.

	Median # of previous TACE	n	Ongoing		Discontinuation due to PD		Discontinuati on due to AE		Other	
Child-Pugh A	2	76	45	59.2%	10	13.2%	18	23.7%	3	3.9%
5A, ALBI Grade1	1	27	18	66.7%	4	14.8%	3	11.1%	2	7.4%
5A, ALBI Grade2	2	19	10	52.6%	3	15.8%	5	26.3%	1	5.3%
6A	4	30	17	56.7%	3	10.0%	10	33.3%	0	0.0%
Child-Pugh B	7	6	0	0.0%	0	0.0%	6	100.0%	0	0.0%
Total	2	82	45	54.9%	10	12.2%	24	29.3%	3	3.7%

PD: progressive disease, AE: adverse event.

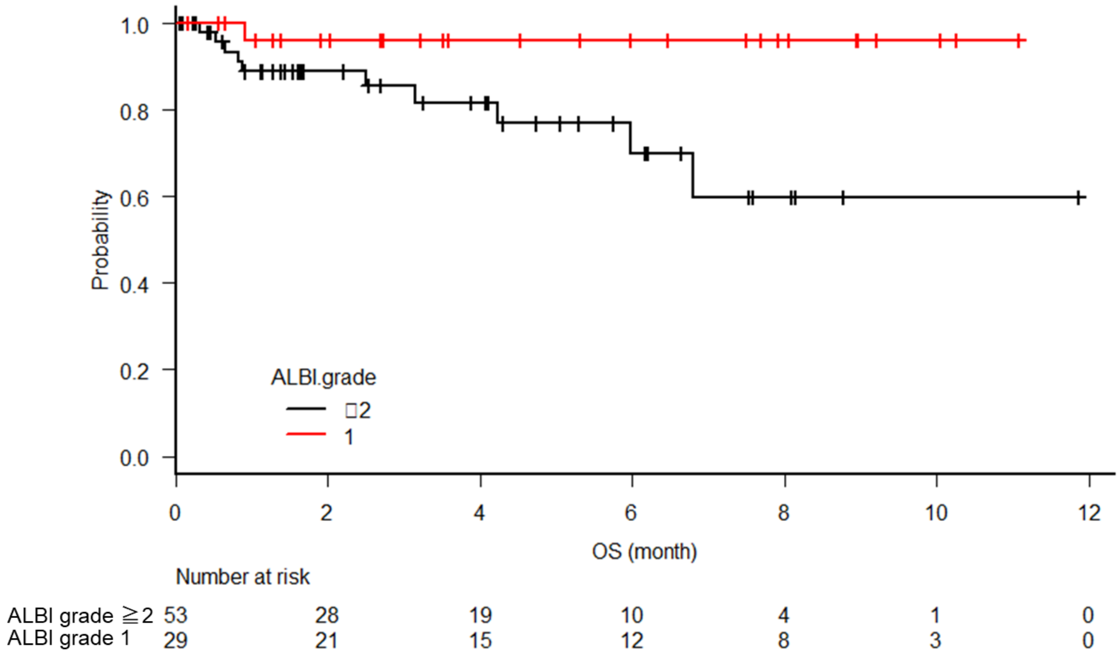

Figure S1. Kaplan–Meier Estimates of OS in Patients Treated with Lenvatinib. According to the ALBI Grade.
